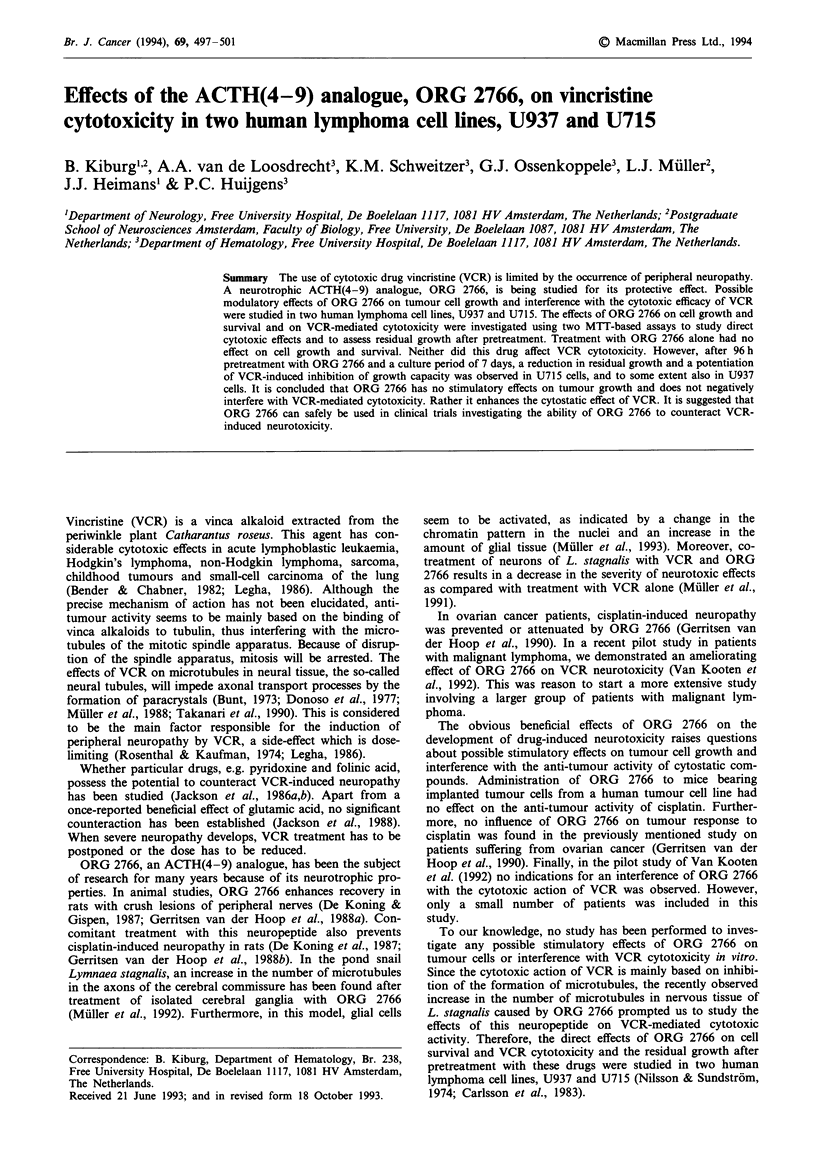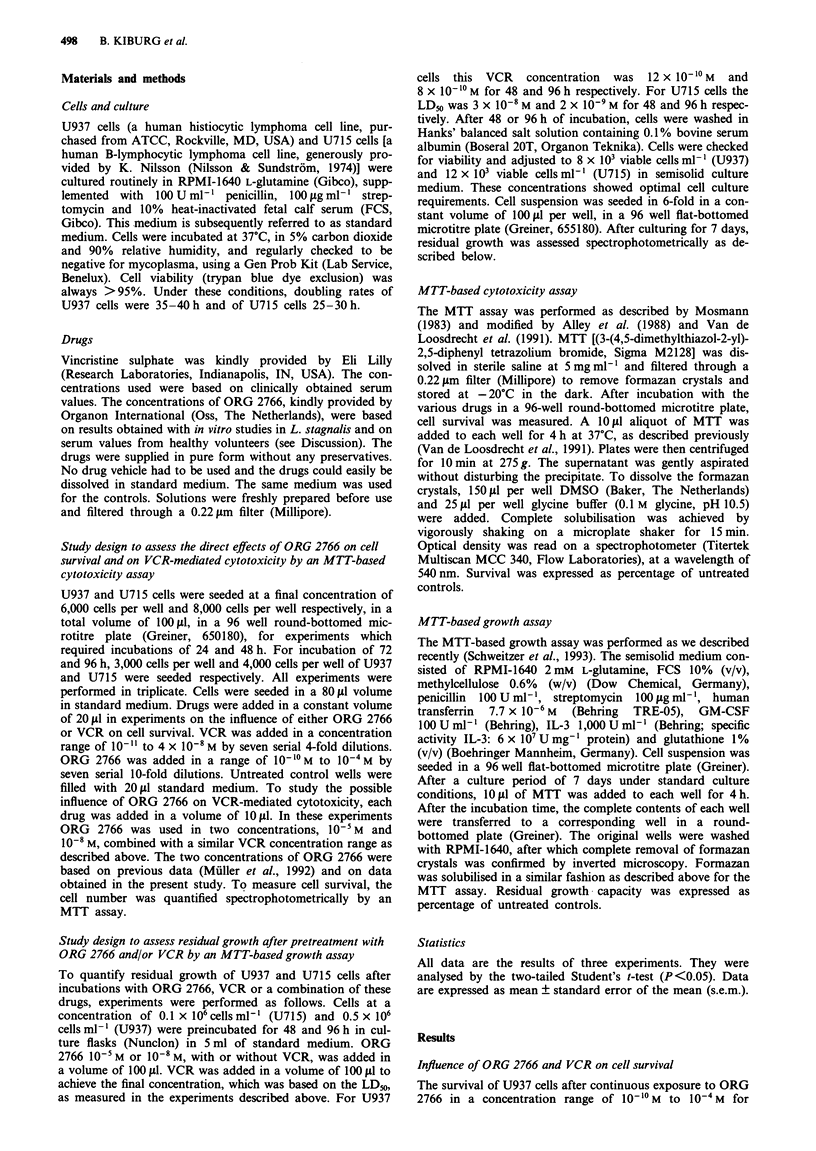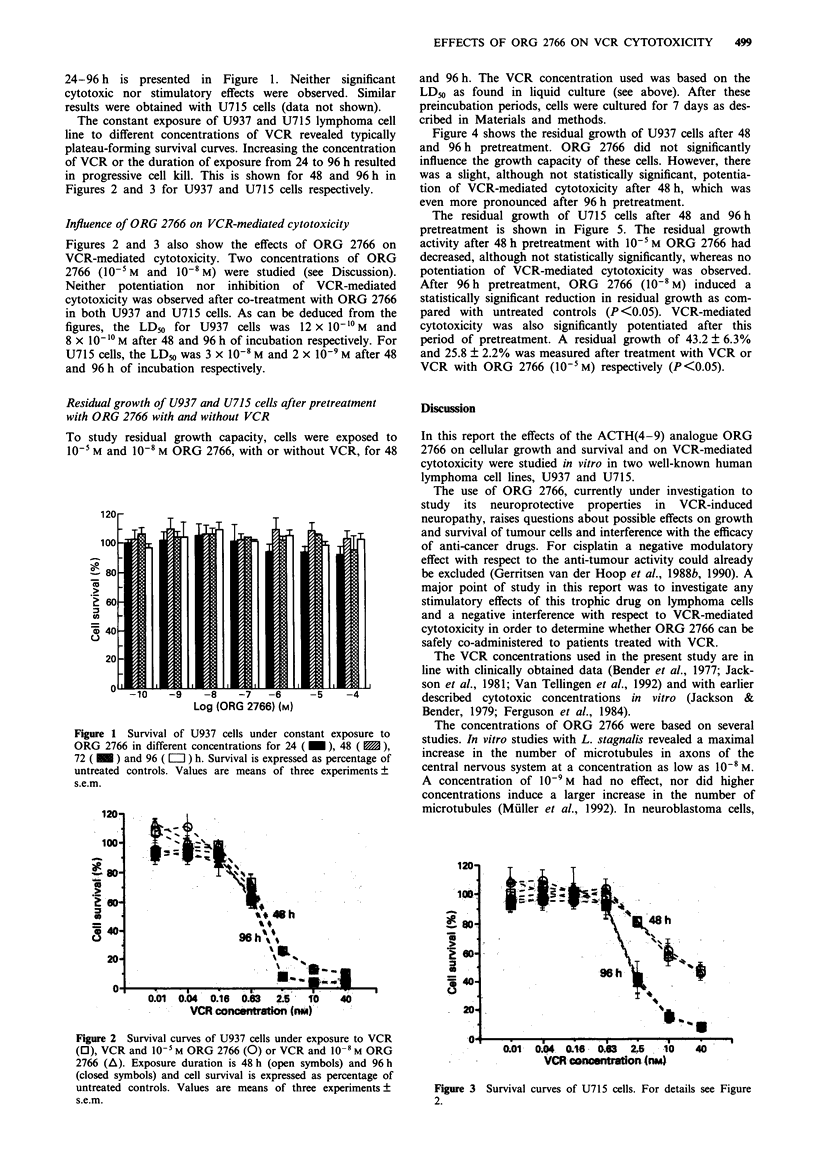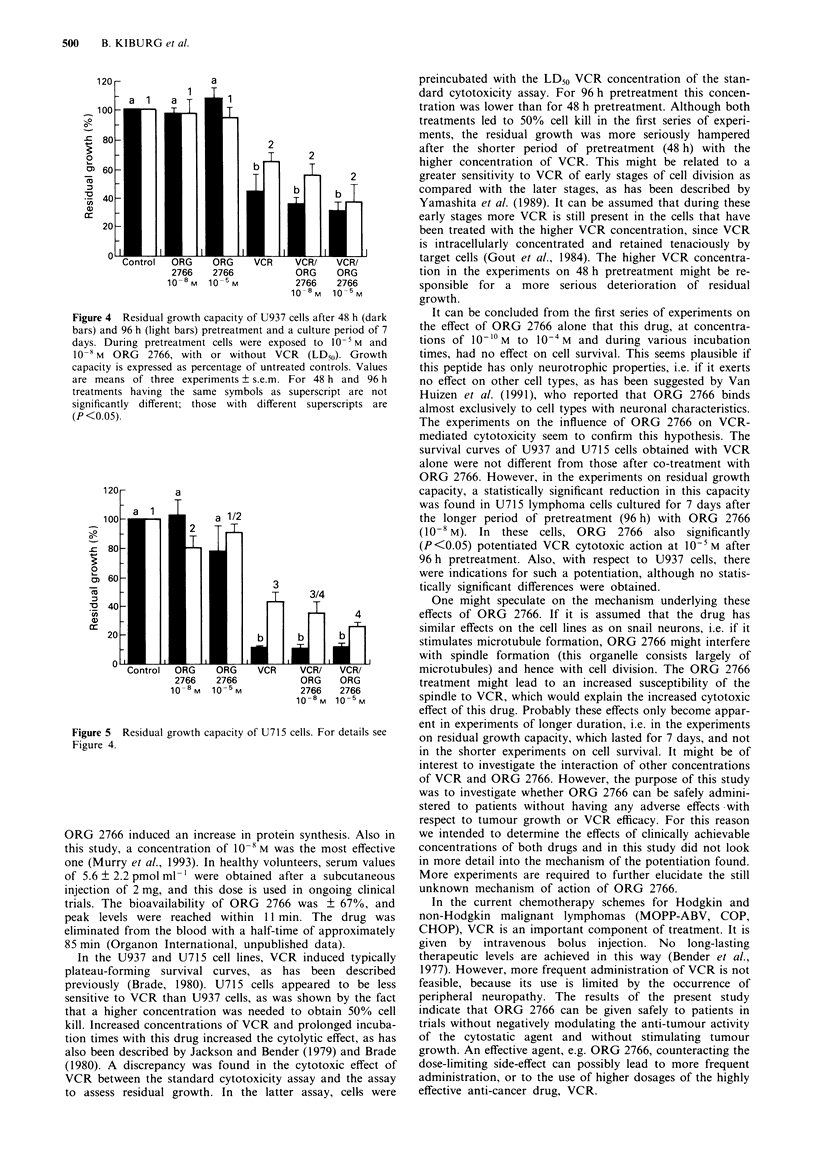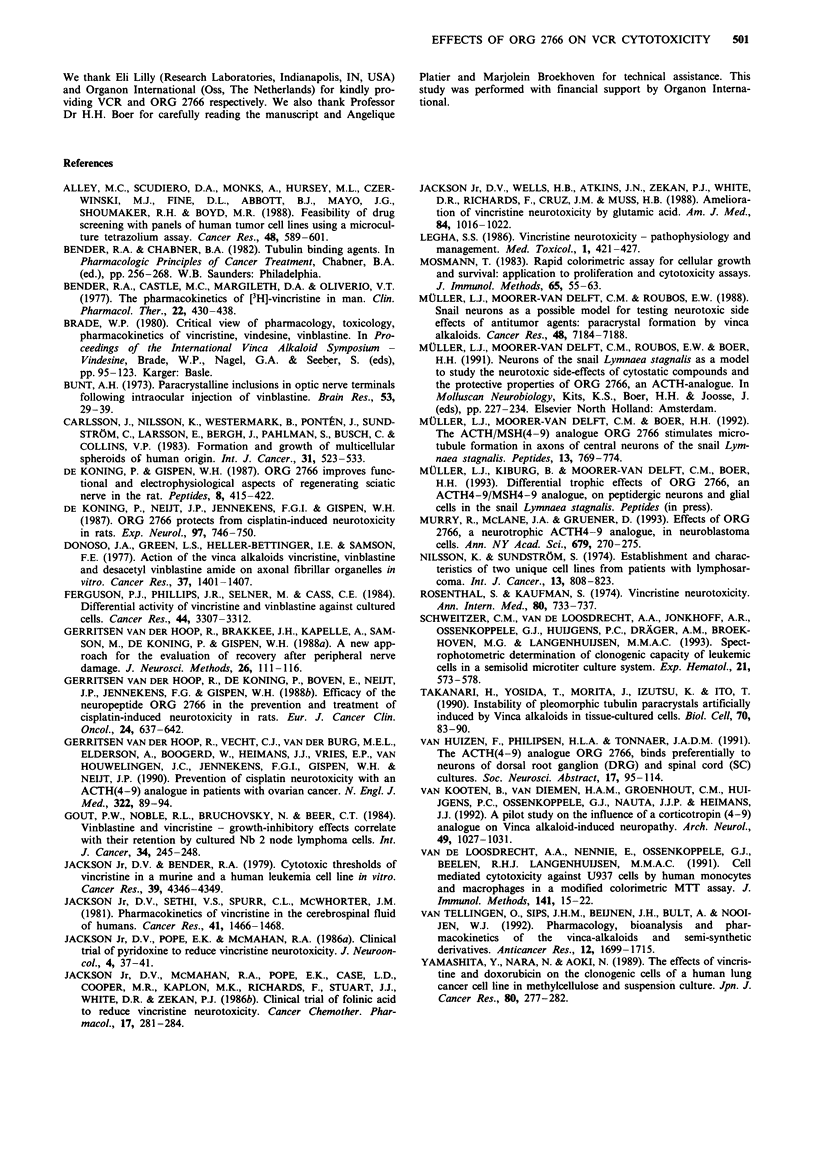# Effects of the ACTH(4-9) analogue, ORG 2766, on vincristine cytotoxicity in two human lymphoma cell lines, U937 and U715.

**DOI:** 10.1038/bjc.1994.90

**Published:** 1994-03

**Authors:** B. Kiburg, A. A. van de Loosdrecht, K. M. Schweitzer, G. J. Ossenkoppele, L. J. Müller, J. J. Heimans, P. C. Huijgens

**Affiliations:** Department of Neurology, Free University Hospital, Amsterdam, The Netherlands.

## Abstract

The use of cytotoxic drug vincristine (VCR) is limited by the occurrence of peripheral neuropathy. A neurotrophic ACTH(4-9) analogue, ORG 2766, is being studied for its protective effect. Possible modulatory effects of ORG 2766 on tumour cell growth and interference with the cytotoxic efficacy of VCR were studied in two human lymphoma cell lines, U937 and U715. The effects of ORG 2766 on cell growth and survival and on VCR-mediated cytotoxicity were investigated using two MTT-based assays to study direct cytotoxic effects and to assess residual growth after pretreatment. Treatment with ORG 2766 alone had no effect on cell growth and survival. Neither did this drug affect VCR cytotoxicity. However, after 96 h pretreatment with ORG 2766 and a culture period of 7 days, a reduction in residual growth and a potentiation of VCR-induced inhibition of growth capacity was observed in U715 cells, and to some extent also in U937 cells. It is concluded that ORG 2766 has no stimulatory effects on tumour growth and does not negatively interfere with VCR-mediated cytotoxicity. Rather it enhances the cytostatic effect of VCR. It is suggested that ORG 2766 can safely be used in clinical trials investigating the ability of ORG 2766 to counteract VCR-induced neurotoxicity.


Br. J. Cancer (1994), 69, 497-501 © Macmillan Press Ltd., 1994

### Effects of the ACTH(4-9) analogue, ORG 2766, on vincristine cytotoxicity in two human lymphoma cell lines, U937 and U715

B. Kiburg[1,2], A.A. van de Loosdrecht[3], K.M. Schweitzer[3], G.J. Ossenkoppele[3], L.J. Müller[2], J.J. Heimans[1] & P.C. Huijgens[3]

[1]*Department of Neurology, Free University Hospital, De Boelelaan 1117, 1081 HV Amsterdam, The Netherlands;* [2]*Postgraduate School of Neurosciences Amsterdam, Faculty of Biology, Free University, De Boelelaan 1087, 1081 HV Amsterdam, The Netherlands;* [3]*Department of Hematology, Free University Hospital, De Boelelaan 1117, 1081 HV Amsterdam, The Netherlands.*

**Summary** The use of cytotoxic drug vincristine (VCR) is limited by the occurrence of peripheral neuropathy. A neurotrophic ACTH(4-9) analogue, ORG 2766, is being studied for its protective effect. Possible modulatory effects of ORG 2766 on tumour cell growth and interference with the cytotoxic efficacy of VCR were studied in two human lymphoma cell lines, U937 and U715. The effects of ORG 2766 on cell growth and survival and on VCR-mediated cytotoxicity were investigated using two MTT-based assays to study direct cytotoxic effects and to assess residual growth after pretreatment. Treatment with ORG 2766 alone had no effect on cell growth and survival. Neither did this drug affect VCR cytotoxicity. However, after 96 h pretreatment with ORG 2766 and a culture period of 7 days, a reduction in residual growth and a potentiation of VCR-induced inhibition of growth capacity was observed in U715 cells, and to some extent also in U937 cells. It is concluded that ORG 2766 has no stimulatory effects on tumour growth and does not negatively interfere with VCR-mediated cytotoxicity. Rather it enhances the cytostatic effect of VCR. It is suggested that ORG 2766 can safely be used in clinical trials investigating the ability of ORG 2766 to counteract VCR-induced neurotoxicity.

Vincristine (VCR) is a vinca alkaloid extracted from the periwinkle plant *Catharantus roseus*. This agent has considerable cytotoxic effects in acute lymphoblastic leukaemia, Hodgkin's lymphoma, non-Hodgkin lymphoma, sarcoma, childhood tumours and small-cell carcinoma of the lung (Bender & Chabner, 1982; Legha, 1986). Although the precise mechanism of action has not been elucidated, anti-tumour activity seems to be mainly based on the binding of vinca alkaloids to tubulin, thus interfering with the microtubules of the mitotic spindle apparatus. Because of disruption of the spindle apparatus, mitosis will be arrested. The effects of VCR on microtubules in neural tissue, the so-called neural tubules, will impede axonal transport processes by the formation of paracrystals (Bunt, 1973; Donoso *et al.*, 1977; Müller *et al.*, 1988; Takanari *et al.*, 1990). This is considered to be the main factor responsible for the induction of peripheral neuropathy by VCR, a side-effect which is dose-limiting (Rosenthal & Kaufman, 1974; Legha, 1986).

Whether particular drugs, e.g. pyridoxine and folinic acid, possess the potential to counteract VCR-induced neuropathy has been studied (Jackson *et al.*, 1986*a*,*b*). Apart from a once-reported beneficial effect of glutamic acid, no significant counteraction has been established (Jackson *et al.*, 1988). When severe neuropathy develops, VCR treatment has to be postponed or the dose has to be reduced.

ORG 2766, an ACTH(4-9) analogue, has been the subject of research for many years because of its neurotrophic properties. In animal studies, ORG 2766 enhances recovery in rats with crush lesions of peripheral nerves (De Koning & Gispen, 1987; Gerritsen van der Hoop *et al.*, 1988*a*). Concomitant treatment with this neuropeptide also prevents cisplatin-induced neuropathy in rats (De Koning *et al.*, 1987; Gerritsen van der Hoop *et al.*, 1988*b*). In the pond snail *Lymnaea stagnalis*, an increase in the number of microtubules in the axons of the cerebral commissure has been found after treatment of isolated cerebral ganglia with ORG 2766 (Müller *et al.*, 1992). Furthermore, in this model, glial cells seem to be activated, as indicated by a change in the chromatin pattern in the nuclei and an increase in the amount of glial tissue (Müller *et al.*, 1993). Moreover, co-treatment of neurons of *L. stagnalis* with VCR and ORG 2766 results in a decrease in the severity of neurotoxic effects as compared with treatment with VCR alone (Müller *et al.*, 1991).

In ovarian cancer patients, cisplatin-induced neuropathy was prevented or attenuated by ORG 2766 (Gerritsen van der Hoop *et al.*, 1990). In a recent pilot study in patients with malignant lymphoma, we demonstrated an ameliorating effect of ORG 2766 on VCR neurotoxicity (Van Kooten *et al.*, 1992). This was reason to start a more extensive study involving a larger group of patients with malignant lymphoma.

The obvious beneficial effects of ORG 2766 on the development of drug-induced neurotoxicity raises questions about possible stimulatory effects on tumour cell growth and interference with the anti-tumour activity of cytostatic compounds. Administration of ORG 2766 to mice bearing implanted tumour cells from a human tumour cell line had no effect on the anti-tumour activity of cisplatin. Furthermore, no influence of ORG 2766 on tumour response to cisplatin was found in the previously mentioned study on patients suffering from ovarian cancer (Gerritsen van der Hoop *et al.*, 1990). Finally, in the pilot study of Van Kooten *et al.* (1992) no indications for an interference of ORG 2766 with the cytotoxic action of VCR was observed. However, only a small number of patients was included in this study.

To our knowledge, no study has been performed to investigate any possible stimulatory effects of ORG 2766 on tumour cells or interference with VCR cytotoxicity *in vitro*. Since the cytotoxic action of VCR is mainly based on inhibition of the formation of microtubules, the recently observed increase in the number of microtubules in nervous tissue of *L. stagnalis* caused by ORG 2766 prompted us to study the effects of this neuropeptide on VCR-mediated cytotoxic activity. Therefore, the direct effects of ORG 2766 on cell survival and VCR cytotoxicity and the residual growth after pretreatment with these drugs were studied in two human lymphoma cell lines, U937 and U715 (Nilsson & Sundström, 1974; Carlsson *et al.*, 1983).

Correspondence: B. Kiburg, Department of Hematology, Br. 238, Free University Hospital, De Boelelaan 1117, 1081 HV Amsterdam, The Netherlands.
Received 21 June 1993; and in revised form 18 October 1993.

### Materials and methods

*Cells and culture*

U937 cells (a human histiocytic lymphoma cell line, purchased from ATCC, Rockville, MD, USA) and U715 cells [a human B-lymphocytic lymphoma cell line, generously provided by K. Nilsson (Nilsson & Sundström, 1974)] were cultured routinely in RPMI-1640 L-glutamine (Gibco), supplemented with 100 U $ml^{-1}$ penicillin, 100 $\mu g$ $ml^{-1}$ streptomycin and 10% heat-inactivated fetal calf serum (FCS, Gibco). This medium is subsequently referred to as standard medium. Cells were incubated at 37°C, in 5% carbon dioxide and 90% relative humidity, and regularly checked to be negative for mycoplasma, using a Gen Prob Kit (Lab Service, Benelux). Cell viability (trypan blue dye exclusion) was always >95%. Under these conditions, doubling rates of U937 cells were 35–40 h and of U715 cells 25–30 h.

*Drugs*

Vincristine sulphate was kindly provided by Eli Lilly (Research Laboratories, Indianapolis, IN, USA). The concentrations used were based on clinically obtained serum values. The concentrations of ORG 2766, kindly provided by Organon International (Oss, The Netherlands), were based on results obtained with *in vitro* studies in *L. stagnalis* and on serum values from healthy volunteers (see Discussion). The drugs were supplied in pure form without any preservatives. No drug vehicle had to be used and the drugs could easily be dissolved in standard medium. The same medium was used for the controls. Solutions were freshly prepared before use and filtered through a 0.22 $\mu m$ filter (Millipore).

*Study design to assess the direct effects of ORG 2766 on cell survival and on VCR-mediated cytotoxicity by an MTT-based cytotoxicity assay*

U937 and U715 cells were seeded at a final concentration of 6,000 cells per well and 8,000 cells per well respectively, in a total volume of 100 $\mu l$, in a 96 well round-bottomed microtitre plate (Greiner, 650180), for experiments which required incubations of 24 and 48 h. For incubation of 72 and 96 h, 3,000 cells per well and 4,000 cells per well of U937 and U715 were seeded respectively. All experiments were performed in triplicate. Cells were seeded in a 80 $\mu l$ volume in standard medium. Drugs were added in a constant volume of 20 $\mu l$ in experiments on the influence of either ORG 2766 or VCR on cell survival. VCR was added in a concentration range of $10^{-11}$ to $4 \times 10^{-8}$ M by seven serial 4-fold dilutions. ORG 2766 was added in a range of $10^{-10}$ M to $10^{-4}$ M by seven serial 10-fold dilutions. Untreated control wells were filled with 20 $\mu l$ standard medium. To study the possible influence of ORG 2766 on VCR-mediated cytotoxicity, each drug was added in a volume of 10 $\mu l$. In these experiments ORG 2766 was used in two concentrations, $10^{-5}$ M and $10^{-8}$ M, combined with a similar VCR concentration range as described above. The two concentrations of ORG 2766 were based on previous data (Müller *et al.*, 1992) and on data obtained in the present study. To measure cell survival, the cell number was quantified spectrophotometrically by an MTT assay.

*Study design to assess residual growth after pretreatment with ORG 2766 and/or VCR by an MTT-based growth assay*

To quantify residual growth of U937 and U715 cells after incubations with ORG 2766, VCR or a combination of these drugs, experiments were performed as follows. Cells at a concentration of $0.1 \times 10^6$ cells $ml^{-1}$ (U715) and $0.5 \times 10^6$ cells $ml^{-1}$ (U937) were preincubated for 48 and 96 h in culture flasks (Nunclon) in 5 ml of standard medium. ORG 2766 $10^{-5}$ M or $10^{-8}$ M, with or without VCR, was added in a volume of 100 $\mu l$. VCR was added in a volume of 100 $\mu l$ to achieve the final concentration, which was based on the $LD_{50}$, as measured in the experiments described above. For U937 cells this VCR concentration was $12 \times 10^{-10}$ M and $8 \times 10^{-10}$ M for 48 and 96 h respectively. For U715 cells the $LD_{50}$ was $3 \times 10^{-8}$ M and $2 \times 10^{-9}$ M for 48 and 96 h respectively. After 48 or 96 h of incubation, cells were washed in Hanks' balanced salt solution containing 0.1% bovine serum albumin (Boseral 20T, Organon Teknika). Cells were checked for viability and adjusted to $8 \times 10^3$ viable cells $ml^{-1}$ (U937) and $12 \times 10^3$ viable cells $ml^{-1}$ (U715) in semisolid culture medium. These concentrations showed optimal cell culture requirements. Cell suspension was seeded in 6-fold in a constant volume of 100 $\mu l$ per well, in a 96 well flat-bottomed microtitre plate (Greiner, 655180). After culturing for 7 days, residual growth was assessed spectrophotometrically as described below.

*MTT-based cytotoxicity assay*

The MTT assay was performed as described by Mosmann (1983) and modified by Alley *et al.* (1988) and Van de Loosdrecht *et al.* (1991). MTT [(3-(4,5-dimethylthiazol-2-yl)-2,5-diphenyl tetrazolium bromide, Sigma M2128] was dissolved in sterile saline at 5 mg $ml^{-1}$ and filtered through a 0.22 $\mu m$ filter (Millipore) to remove formazan crystals and stored at −20°C in the dark. After incubation with the various drugs in a 96-well round-bottomed microtitre plate, cell survival was measured. A 10 $\mu l$ aliquot of MTT was added to each well for 4 h at 37°C, as described previously (Van de Loosdrecht *et al.*, 1991). Plates were then centrifuged for 10 min at 275 *g*. The supernatant was gently aspirated without disturbing the precipitate. To dissolve the formazan crystals, 150 $\mu l$ per well DMSO (Baker, The Netherlands) and 25 $\mu l$ per well glycine buffer (0.1 M glycine, pH 10.5) were added. Complete solubilisation was achieved by vigorously shaking on a microplate shaker for 15 min. Optical density was read on a spectrophotometer (Titertek Multiscan MCC 340, Flow Laboratories), at a wavelength of 540 nm. Survival was expressed as percentage of untreated controls.

*MTT-based growth assay*

The MTT-based growth assay was performed as we described recently (Schweitzer *et al.*, 1993). The semisolid medium consisted of RPMI-1640 2 mM L-glutamine, FCS 10% (v/v), methylcellulose 0.6% (w/v) (Dow Chemical, Germany), penicillin 100 U $ml^{-1}$, streptomycin 100 $\mu g$ $ml^{-1}$, human transferrin $7.7 \times 10^{-6}$ M (Behring TRE-05), GM-CSF 100 U $ml^{-1}$ (Behring), IL-3 1,000 U $ml^{-1}$ (Behring; specific activity IL-3: $6 \times 10^7$ U $mg^{-1}$ protein) and glutathione 1% (v/v) (Boehringer Mannheim, Germany). Cell suspension was seeded in a 96 well flat-bottomed microtitre plate (Greiner). After a culture period of 7 days under standard culture conditions, 10 $\mu l$ of MTT was added to each well for 4 h. After the incubation time, the complete contents of each well were transferred to a corresponding well in a round-bottomed plate (Greiner). The original wells were washed with RPMI-1640, after which complete removal of formazan crystals was confirmed by inverted microscopy. Formazan was solubilised in a similar fashion as described above for the MTT assay. Residual growth capacity was expressed as percentage of untreated controls.

*Statistics*

All data are the results of three experiments. They were analysed by the two-tailed Student's *t*-test ($P<0.05$). Data are expressed as mean ± standard error of the mean (s.e.m.).

### Results

*Influence of ORG 2766 and VCR on cell survival*

The survival of U937 cells after continuous exposure to ORG 2766 in a concentration range of $10^{-10}$ M to $10^{-4}$ M for

24–96 h is presented in Figure 1. Neither significant cytotoxic nor stimulatory effects were observed. Similar results were obtained with U715 cells (data not shown).

The constant exposure of U937 and U715 lymphoma cell line to different concentrations of VCR revealed typically plateau-forming survival curves. Increasing the concentration of VCR or the duration of exposure from 24 to 96 h resulted in progressive cell kill. This is shown for 48 and 96 h in Figures 2 and 3 for U937 and U715 cells respectively.

*Influence of ORG 2766 on VCR-mediated cytotoxicity*

Figures 2 and 3 also show the effects of ORG 2766 on VCR-mediated cytotoxicity. Two concentrations of ORG 2766 ($10^{-5}$ M and $10^{-8}$ M) were studied (see Discussion). Neither potentiation nor inhibition of VCR-mediated cytotoxicity was observed after co-treatment with ORG 2766 in both U937 and U715 cells. As can be deduced from the figures, the $LD_{50}$ for U937 cells was $12 \times 10^{-10}$ M and $8 \times 10^{-10}$ M after 48 and 96 h of incubation respectively. For U715 cells, the $LD_{50}$ was $3 \times 10^{-8}$ M and $2 \times 10^{-9}$ M after 48 and 96 h of incubation respectively.

*Residual growth of U937 and U715 cells after pretreatment with ORG 2766 with and without VCR*

To study residual growth capacity, cells were exposed to $10^{-5}$ M and $10^{-8}$ M ORG 2766, with or without VCR, for 48 and 96 h. The VCR concentration used was based on the $LD_{50}$ as found in liquid culture (see above). After these preincubation periods, cells were cultured for 7 days as described in Materials and methods.

Figure 4 shows the residual growth of U937 cells after 48 and 96 h pretreatment. ORG 2766 did not significantly influence the growth capacity of these cells. However, there was a slight, although not statistically significant, potentiation of VCR-mediated cytotoxicity after 48 h, which was even more pronounced after 96 h pretreatment.

The residual growth of U715 cells after 48 and 96 h pretreatment is shown in Figure 5. The residual growth activity after 48 h pretreatment with $10^{-5}$ M ORG 2766 had decreased, although not statistically significantly, whereas no potentiation of VCR-mediated cytotoxicity was observed. After 96 h pretreatment, ORG 2766 ($10^{-8}$ M) induced a statistically significant reduction in residual growth as compared with untreated controls ($P<0.05$). VCR-mediated cytotoxicity was also significantly potentiated after this period of pretreatment. A residual growth of $43.2 \pm 6.3\%$ and $25.8 \pm 2.2\%$ was measured after treatment with VCR or VCR with ORG 2766 ($10^{-5}$ M) respectively ($P<0.05$).

**Figure 1** Survival of U937 cells under constant exposure to ORG 2766 in different concentrations for 24 (■), 48 (▨), 72 (▩) and 96 (□) h. Survival is expressed as percentage of untreated controls. Values are means of three experiments ± s.e.m.

**Figure 2** Survival curves of U937 cells under exposure to VCR (□), VCR and $10^{-5}$ M ORG 2766 (○) or VCR and $10^{-8}$ M ORG 2766 (△). Exposure duration is 48 h (open symbols) and 96 h (closed symbols) and cell survival is expressed as percentage of untreated controls. Values are means of three experiments ± s.e.m.

### Discussion

In this report the effects of the ACTH(4–9) analogue ORG 2766 on cellular growth and survival and on VCR-mediated cytotoxicity were studied *in vitro* in two well-known human lymphoma cell lines, U937 and U715.

The use of ORG 2766, currently under investigation to study its neuroprotective properties in VCR-induced neuropathy, raises questions about possible effects on growth and survival of tumour cells and interference with the efficacy of anti-cancer drugs. For cisplatin a negative modulatory effect with respect to the anti-tumour activity could already be excluded (Gerritsen van der Hoop *et al.*, 1988*b*, 1990). A major point of study in this report was to investigate any stimulatory effects of this trophic drug on lymphoma cells and a negative interference with respect to VCR-mediated cytotoxicity in order to determine whether ORG 2766 can be safely co-administered to patients treated with VCR.

The VCR concentrations used in the present study are in line with clinically obtained data (Bender *et al.*, 1977; Jackson *et al.*, 1981; Van Tellingen *et al.*, 1992) and with earlier described cytotoxic concentrations *in vitro* (Jackson & Bender, 1979; Ferguson *et al.*, 1984).

The concentrations of ORG 2766 were based on several studies. *In vitro* studies with *L. stagnalis* revealed a maximal increase in the number of microtubules in axons of the central nervous system at a concentration as low as $10^{-8}$ M. A concentration of $10^{-9}$ M had no effect, nor did higher concentrations induce a larger increase in the number of microtubules (Müller *et al.*, 1992). In neuroblastoma cells,

**Figure 3** Survival curves of U715 cells. For details see Figure 2.

**Figure 4** Residual growth capacity of U937 cells after 48 h (dark bars) and 96 h (light bars) pretreatment and a culture period of 7 days. During pretreatment cells were exposed to $10^{-5}$ M and $10^{-8}$ M ORG 2766, with or without VCR ($LD_{50}$). Growth capacity is expressed as percentage of untreated controls. Values are means of three experiments ± s.e.m. For 48 h and 96 h treatments having the same symbols as superscript are not significantly different; those with different superscripts are ($P<0.05$).

**Figure 5** Residual growth capacity of U715 cells. For details see Figure 4.

ORG 2766 induced an increase in protein synthesis. Also in this study, a concentration of $10^{-8}$ M was the most effective one (Murry *et al.*, 1993). In healthy volunteers, serum values of 5.6 ± 2.2 pmol $ml^{-1}$ were obtained after a subcutaneous injection of 2 mg, and this dose is used in ongoing clinical trials. The bioavailability of ORG 2766 was ± 67%, and peak levels were reached within 11 min. The drug was eliminated from the blood with a half-time of approximately 85 min (Organon International, unpublished data).

In the U937 and U715 cell lines, VCR induced typically plateau-forming survival curves, as has been described previously (Brade, 1980). U715 cells appeared to be less sensitive to VCR than U937 cells, as was shown by the fact that a higher concentration was needed to obtain 50% cell kill. Increased concentrations of VCR and prolonged incubation times with this drug increased the cytolytic effect, as has also been described by Jackson and Bender (1979) and Brade (1980). A discrepancy was found in the cytotoxic effect of VCR between the standard cytotoxicity assay and the assay to assess residual growth. In the latter assay, cells were preincubated with the $LD_{50}$ VCR concentration of the standard cytotoxicity assay. For 96 h pretreatment this concentration was lower than for 48 h pretreatment. Although both treatments led to 50% cell kill in the first series of experiments, the residual growth was more seriously hampered after the shorter period of pretreatment (48 h) with the higher concentration of VCR. This might be related to a greater sensitivity to VCR of early stages of cell division as compared with the later stages, as has been described by Yamashita *et al.* (1989). It can be assumed that during these early stages more VCR is still present in the cells that have been treated with the higher VCR concentration, since VCR is intracellularly concentrated and retained tenaciously by target cells (Gout *et al.*, 1984). The higher VCR concentration in the experiments on 48 h pretreatment might be responsible for a more serious deterioration of residual growth.

It can be concluded from the first series of experiments on the effect of ORG 2766 alone that this drug, at concentrations of $10^{-10}$ M to $10^{-4}$ M and during various incubation times, had no effect on cell survival. This seems plausible if this peptide has only neurotrophic properties, i.e. if it exerts no effect on other cell types, as has been suggested by Van Huizen *et al.* (1991), who reported that ORG 2766 binds almost exclusively to cell types with neuronal characteristics. The experiments on the influence of ORG 2766 on VCR-mediated cytotoxicity seem to confirm this hypothesis. The survival curves of U937 and U715 cells obtained with VCR alone were not different from those after co-treatment with ORG 2766. However, in the experiments on residual growth capacity, a statistically significant reduction in this capacity was found in U715 lymphoma cells cultured for 7 days after the longer period of pretreatment (96 h) with ORG 2766 ($10^{-8}$ M). In these cells, ORG 2766 also significantly ($P<0.05$) potentiated VCR cytotoxic action at $10^{-5}$ M after 96 h pretreatment. Also, with respect to U937 cells, there were indications for such a potentiation, although no statistically significant differences were obtained.

One might speculate on the mechanism underlying these effects of ORG 2766. If it is assumed that the drug has similar effects on the cell lines as on snail neurons, i.e. if it stimulates microtubule formation, ORG 2766 might interfere with spindle formation (this organelle consists largely of microtubules) and hence with cell division. The ORG 2766 treatment might lead to an increased susceptibility of the spindle to VCR, which would explain the increased cytotoxic effect of this drug. Probably these effects only become apparent in experiments of longer duration, i.e. in the experiments on residual growth capacity, which lasted for 7 days, and not in the shorter experiments on cell survival. It might be of interest to investigate the interaction of other concentrations of VCR and ORG 2766. However, the purpose of this study was to investigate whether ORG 2766 can be safely administered to patients without having any adverse effects with respect to tumour growth or VCR efficacy. For this reason we intended to determine the effects of clinically achievable concentrations of both drugs and in this study did not look in more detail into the mechanism of the potentiation found. More experiments are required to further elucidate the still unknown mechanism of action of ORG 2766.

In the current chemotherapy schemes for Hodgkin and non-Hodgkin malignant lymphomas (MOPP-ABV, COP, CHOP), VCR is an important component of treatment. It is given by intravenous bolus injection. No long-lasting therapeutic levels are achieved in this way (Bender *et al.*, 1977). However, more frequent administration of VCR is not feasible, because its use is limited by the occurrence of peripheral neuropathy. The results of the present study indicate that ORG 2766 can be given safely to patients in trials without negatively modulating the anti-tumour activity of the cytostatic agent and without stimulating tumour growth. An effective agent, e.g. ORG 2766, counteracting the dose-limiting side-effect can possibly lead to more frequent administration, or to the use of higher dosages of the highly effective anti-cancer drug, VCR.

We thank Eli Lilly (Research Laboratories, Indianapolis, IN, USA) and Organon International (Oss, The Netherlands) for kindly providing VCR and ORG 2766 respectively. We also thank Professor Dr H.H. Boer for carefully reading the manuscript and Angelique Platier and Marjolein Broekhoven for technical assistance. This study was performed with financial support by Organon International.

### References

ALLEY, M.C., SCUDIERO, D.A., MONKS, A., HURSEY, M.L., CZERWINSKI, M.J., FINE, D.L., ABBOTT, B.J., MAYO, J.G., SHOUMAKER, R.H. & BOYD, M.R. (1988). Feasibility of drug screening with panels of human tumor cell lines using a microculture tetrazolium assay. *Cancer Res.*, **48**, 589–601.

BENDER, R.A. & CHABNER, B.A. (1982). Tubulin binding agents. In *Pharmacologic Principles of Cancer Treatment*, Chabner, B.A. (ed.), pp. 256–268. W.B. Saunders: Philadelphia.

BENDER, R.A., CASTLE, M.C., MARGILETH, D.A. & OLIVERIO, V.T. (1977). The pharmacokinetics of [$^3$H]-vincristine in man. *Clin. Pharmacol. Ther.*, **22**, 430–438.

BRADE, W.P. (1980). Critical view of pharmacology, toxicology, pharmacokinetics of vincristine, vindesine, vinblastine. In *Proceedings of the International Vinca Alkaloid Symposium – Vindesine*, Brade, W.P., Nagel, G.A. & Seeber, S. (eds), pp. 95–123. Karger: Basle.

BUNT, A.H. (1973). Paracrystalline inclusions in optic nerve terminals following intraocular injection of vinblastine. *Brain Res.*, **53**, 29–39.

CARLSSON, J., NILSSON, K., WESTERMARK, B., PONTÉN, J., SUNDSTRÖM, C., LARSSON, E., BERGH, J., PAHLMAN, S., BUSCH, C. & COLLINS, V.P. (1983). Formation and growth of multicellular spheroids of human origin. *Int. J. Cancer.*, **31**, 523–533.

DE KONING, P. & GISPEN, W.H. (1987). ORG 2766 improves functional and electrophysiological aspects of regenerating sciatic nerve in the rat. *Peptides*, **8**, 415–422.

DE KONING, P., NEIJT, J.P., JENNEKENS, F.G.I. & GISPEN, W.H. (1987). ORG 2766 protects from cisplatin-induced neurotoxicity in rats. *Exp. Neurol.*, **97**, 746–750.

DONOSO, J.A., GREEN, L.S., HELLER-BETTINGER, I.E. & SAMSON, F.E. (1977). Action of the vinca alkaloids vincristine, vinblastine and desacetyl vinblastine amide on axonal fibrillar organelles *in vitro*. *Cancer Res.*, **37**, 1401–1407.

FERGUSON, P.J., PHILLIPS, J.R., SELNER, M. & CASS, C.E. (1984). Differential activity of vincristine and vinblastine against cultured cells. *Cancer Res.*, **44**, 3307–3312.

GERRITSEN VAN DER HOOP, R., BRAKKEE, J.H., KAPELLE, A., SAMSON, M., DE KONING, P. & GISPEN, W.H. (1988*a*). A new approach for the evaluation of recovery after peripheral nerve damage. *J. Neurosci. Methods*, **26**, 111–116.

GERRITSEN VAN DER HOOP, R., DE KONING, P., BOVEN, E., NEIJT, J.P., JENNEKENS, F.G. & GISPEN, W.H. (1988*b*). Efficacy of the neuropeptide ORG 2766 in the prevention and treatment of cisplatin-induced neurotoxicity in rats. *Eur. J. Cancer Clin. Oncol.*, **24**, 637–642.

GERRITSEN VAN DER HOOP, R., VECHT, C.J., VAN DER BURG, M.E.L., ELDERSON, A., BOOGERD, W., HEIMANS, J.J., VRIES, E.P., VAN HOUWELINGEN, J.C., JENNEKENS, F.G.I., GISPEN, W.H. & NEIJT, J.P. (1990). Prevention of cisplatin neurotoxicity with an ACTH(4–9) analogue in patients with ovarian cancer. *N. Engl. J. Med.*, **322**, 89–94.

GOUT, P.W., NOBLE, R.L., BRUCHOVSKY, N. & BEER, C.T. (1984). Vinblastine and vincristine – growth-inhibitory effects correlate with their retention by cultured Nb 2 node lymphoma cells. *Int. J. Cancer*, **34**, 245–248.

JACKSON Jr, D.V. & BENDER, R.A. (1979). Cytotoxic thresholds of vincristine in a murine and a human leukemia cell line *in vitro*. *Cancer Res.*, **39**, 4346–4349.

JACKSON Jr, D.V., SETHI, V.S., SPURR, C.L., MCWHORTER, J.M. (1981). Pharmacokinetics of vincristine in the cerebrospinal fluid of humans. *Cancer Res.*, **41**, 1466–1468.

JACKSON Jr, D.V., POPE, E.K. & MCMAHAN, R.A. (1986*a*). Clinical trial of pyridoxine to reduce vincristine neurotoxicity. *J. Neurooncol.*, **4**, 37–41.

JACKSON Jr, D.V., MCMAHAN, R.A., POPE, E.K., CASE, L.D., COOPER, M.R., KAPLON, M.K., RICHARDS, F., STUART, J.J., WHITE, D.R. & ZEKAN, P.J. (1986*b*). Clinical trial of folinic acid to reduce vincristine neurotoxicity. *Cancer Chemother. Pharmacol.*, **17**, 281–284.

JACKSON Jr, D.V., WELLS, H.B., ATKINS, J.N., ZEKAN, P.J., WHITE, D.R., RICHARDS, F., CRUZ, J.M. & MUSS, H.B. (1988). Amelioration of vincristine neurotoxicity by glutamic acid. *Am. J. Med.*, **84**, 1016–1022.

LEGHA, S.S. (1986). Vincristine neurotoxicity – pathophysiology and management. *Med. Toxicol.*, **1**, 421–427.

MOSMANN, T. (1983). Rapid colorimetric assay for cellular growth and survival: application to proliferation and cytotoxicity assays. *J. Immunol. Methods*, **65**, 55–63.

MÜLLER, L.J., MOORER-VAN DELFT, C.M. & ROUBOS, E.W. (1988). Snail neurons as a possible model for testing neurotoxic side effects of antitumor agents: paracrystal formation by vinca alkaloids. *Cancer Res.*, **48**, 7184–7188.

MÜLLER, L.J., MOORER-VAN DELFT, C.M., ROUBOS, E.W. & BOER, H.H. (1991). Neurons of the snail *Lymnaea stagnalis* as a model to study the neurotoxic side-effects of cytostatic compounds and the protective properties of ORG 2766, an ACTH-analogue. In *Molluscan Neurobiology*, Kits, K.S., Boer, H.H. & Joosse, J. (eds), pp. 227–234. Elsevier North Holland: Amsterdam.

MÜLLER, L.J., MOORER-VAN DELFT, C.M. & BOER, H.H. (1992). The ACTH/MSH(4–9) analogue ORG 2766 stimulates microtubule formation in axons of central neurons of the snail *Lymnaea stagnalis*. *Peptides*, **13**, 769–774.

MÜLLER, L.J., KIBURG, B. & MOORER-VAN DELFT, C.M., BOER, H.H. (1993). Differential trophic effects of ORG 2766, an ACTH4–9/MSH4–9 analogue, on peptidergic neurons and glial cells in the snail *Lymnaea stagnalis*. *Peptides* (in press).

MURRY, R., MCLANE, J.A. & GRUENER, D. (1993). Effects of ORG 2766, a neurotrophic ACTH4–9 analogue, in neuroblastoma cells. *Ann. NY Acad. Sci.*, **679**, 270–275.

NILSSON, K. & SUNDSTRÖM, S. (1974). Establishment and characteristics of two unique cell lines from patients with lymphosarcoma. *Int. J. Cancer.*, **13**, 808–823.

ROSENTHAL, S. & KAUFMAN, S. (1974). Vincristine neurotoxicity. *Ann. Intern. Med.*, **80**, 733–737.

SCHWEITZER, C.M., VAN DE LOOSDRECHT, A.A., JONKHOFF, A.R., OSSENKOPPELE, G.J., HUIJGENS, P.C., DRÄGER, A.M., BROEKHOVEN, M.G. & LANGENHUIJSEN, M.M.A.C. (1993). Spectrophotometric determination of clonogenic capacity of leukemic cells in a semisolid microtiter culture system. *Exp. Hematol.*, **21**, 573–578.

TAKANARI, H., YOSIDA, T., MORITA, J., IZUTSU, K. & ITO, T. (1990). Instability of pleomorphic tubulin paracrystals artificially induced by Vinca alkaloids in tissue-cultured cells. *Biol. Cell*, **70**, 83–90.

VAN HUIZEN, F., PHILIPSEN, H.L.A. & TONNAER, J.A.D.M. (1991). The ACTH(4–9) analogue ORG 2766, binds preferentially to neurons of dorsal root ganglion (DRG) and spinal cord (SC) cultures. *Soc. Neurosci. Abstract*, **17**, 95–114.

VAN KOOTEN, B., VAN DIEMEN, H.A.M., GROENHOUT, C.M., HUIJGENS, P.C., OSSENKOPPELE, G.J., NAUTA, J.J.P. & HEIMANS, J.J. (1992). A pilot study on the influence of a corticotropin (4–9) analogue on Vinca alkaloid-induced neuropathy. *Arch. Neurol.*, **49**, 1027–1031.

VAN DE LOOSDRECHT, A.A., NENNIE, E., OSSENKOPPELE, G.J., BEELEN, R.H.J. LANGENHUIJSEN, M.M.A.C. (1991). Cell mediated cytotoxicity against U937 cells by human monocytes and macrophages in a modified colorimetric MTT assay. *J. Immunol. Methods*, **141**, 15–22.

VAN TELLINGEN, O., SIPS, J.H.M., BEIJNEN, J.H., BULT, A. & NOOIJEN, W.J. (1992). Pharmacology, bioanalysis and pharmacokinetics of the vinca-alkaloids and semi-synthetic derivatives. *Anticancer Res.*, **12**, 1699–1715.

YAMASHITA, Y., NARA, N. & AOKI, N. (1989). The effects of vincristine and doxorubicin on the clonogenic cells of a human lung cancer cell line in methylcellulose and suspension culture. *Jpn. J. Cancer Res.*, **80**, 277–282.